# Preparation of IgY Oriented Conjugated Fe_3_O_4_ MNPs as Immunomagnetic Nanoprobe for Increasing Enrichment Efficiency of *Staphylococcus aureus* Based on Adjusting the pH of the Solution System

**DOI:** 10.3389/fpubh.2022.865828

**Published:** 2022-05-20

**Authors:** Xuening Shi, Hongbin Sun, Hang Li, Shengnan Wei, Jin Jin, Chao Zhao, Juan Wang, Hui Li

**Affiliations:** ^1^School of Public Health, Jilin University, Changchun, China; ^2^China-Japan Union Hospital of Jilin University, Changchun, China

**Keywords:** immunoglobulin yolk (IgY), oriented conjugation, Fe_3_O_4_ magnetic nanoparticles (MNPs), immunomagnetic separation, *Staphylococcus aureus*

## Abstract

Immunomagnetic separation based on Fe_3_O_4_ magnetic nanoparticles (MNPs) has been widely performed in sample pretreatment. The oriented conjugation strategy can achieve a better capture effect than the N-(3-dimethylamlnopropyl)-N'-ethylcarbodiimide hydrochloride (EDC) /N-hydroxysuccinimide (NHS) method. However, immunoglobulin yolk (IgY) cannot be oriented through an SPA strategy like immunoglobulin G (IgG). In this article, an oriented conjugation nanoprobe was prepared for the enrichment of bacteria based on pH adjusting. The main factors affecting the enrichment efficiency were studied, such as the pH of the buffer system, the concentration of IgY, the concentration of nanoprobe, and the enrichment time. Under the optimal conditions, the enrichment efficiency toward target bacteria could reach 92.8%. Combined with PCR, the limit of detection (LOD) was found to be 10^3^ CFU/ml, which was lower than the PCR only. In conclusion, we provided a new protocol for the oriented conjugation of IgY and high sensitivity detection with simple pretreatment.

## Introduction

Food safety is one of the biggest concerns faced worldwide, and it caused about 420,000 deaths worldwide each year (1, 2). In many low- and middle-income countries, healthcare systems are burdened by food-related diseases, and the economic losses due to illness, disability, and death amount to nearly $95 billion, severely hampering economic development (3). Among those, foodborne bacterial infections and diseases have been considered a major threat (4, 5). Rapid and sensitive detection strategies of pathogenic bacteria are a hot topic in the detection field. Until now, many technologies have been applied to bacteria detection, such as the immunofluorescence method (6, 7), nucleic acid amplification technique (8–10), chromometry strategy (11–13), and surface-enhanced Raman spectroscopy(SERS) (14–16). To get higher detection sensitivity, the pretreatment processes were necessary. Therefore, immunomagnetic separation based on Fe_3_O_4_ magnetic nanoparticles (MNPs) had been widely performed in sample pretreatment.

With a high surface-area-to-volume ratio, good dispersibility, and superparamagnetic character (16, 17), MNPs can be easily separated by an external magnetic field (11, 18). Meanwhile, being modified with different functional groups on the surface can improve selectivity. For example, modified MNPs with IgG (19), the MNPs can be used as a capture probe to enrich the target bacteria. *Staphylococcal* protein A (SPA) has a high specific affinity to the Fc portion of IgG (20–22) and can be used in the oriented conjugation of IgG. Besides, the oriented conjugation strategy of IgG developed on this basis has achieved a better capture effect than the traditional EDC/N-hydroxysuccinimide (NHS) method. However, the inherent drawbacks of IgG, such as difficulty to prepare in large quantities, false-positive, and cross-reaction, limited its use in real samples. Besides, the amine/thiol-reactive chemistry conjugation strategies of IgG often resulted in unfavorable outcomes, such as heterogeneous antibody display with hindered biological activity or aggregation (23). Therefore, finding the alternative antibodies of IgG and a new conjugation of strategies is of great significance.

In recent years, immunoglobulin yolk (IgY) has been found to act as an ideal immunological tool in diagnosis and immunotherapy (24). Compared with IgG, it was easy to prepare and can be mass-produced by immunizing laying hens (25, 26). What's more, it did not react with rheumatoid factor, mammalian Fc receptor, and complement (27), and had barely cross-reaction with IgG (28), showing a high specificity. As a result, IgY has a broad application prospect in the development of biological products and the prevention and treatment of diseases. Some researchers have used IgY conjugated with nanoparticles to bacteria detection (29, 30), but the conjugation strategy of these methods was still random conjugation based on the EDC/NHS method. The capture efficiency was unsatisfactory in the practical application. Due to the differences in structure, IgY cannot be oriented to the surface of nanomaterials through the SPA strategy. As compared with random conjugation based on amino-carboxyl reaction, oriented conjugation exposed more binding sites, resulting in significantly increased enrichment efficiency. Thus, it was of great importance to propose an oriented conjugation method for IgY.

**Scheme 1:**
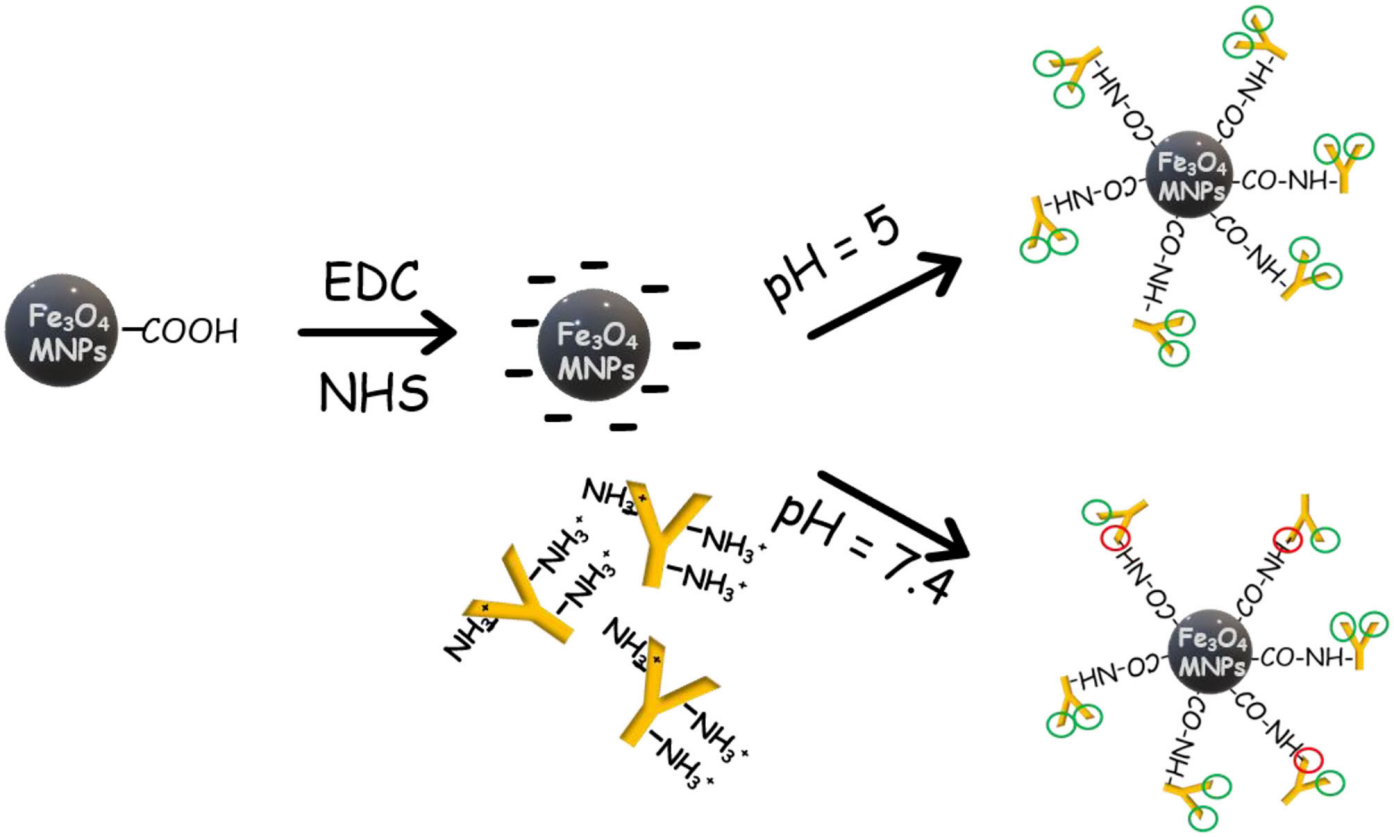
Schematic diagram of the proposed oriented conjugation of immunoglobulin yolk IgY based on adjusting the pH of the buffer system.

Herein, we describe a strategy for oriented conjugation of IgY to Fe_3_O_4_ MNPs based on pH adjusting. As shown in Scheme 1, when the pH of the buffer system was lower than the isoelectric point (pI) of IgY, the charge density of different fragments of IgY presented an uneven distribution state (31). The Fc part of IgY was fully protonated. Based on the electrostatic effect, the IgY was oriented to the surface of the carboxylated Fe_3_O_4_ MNPs through the Fc part and then conjugated to form an immunomagnetic nanoprobe. The enrichment efficiency of the prepared nanoprobe was significantly improved compared with that of the random conjugation method. Taking *Staphylococcus aureus* as a model bacterium, the concentration of nanoprobe and enrichment time was optimized. Finally, the nanoprobe was combined with real-time quantitative PCR and examined in real samples. It was confirmed that the sensitivity was improved in our assay. The nanoprobe synthesized based on our oriented conjugation strategy was efficient, selective, and sensitive for bacteria enrichment in food samples. Besides, the conjugation strategy can be applied in the oriented conjugation of IgY with more materials.

## Materials and Methods

### Reagents

Ferric chloride hexahydrate (FeCl_3_·6H_2_O) and trisodium citrate dihydrate were purchased from Tianjin Damao Chemical Institute (Tianjin China). Sodium acetate and absolute ethyl alcohol were obtained from Beijing Chemical Reagent Company (Beijing, China). Poly(ethylene glycol) (PEG6000) and N-Hydroxysuccinimide (NHS) were bought from Sigma Company (USA). N-(3-dimethylamlnopropyl)-N'-ethylcarbodiimide hydrochloride (EDC) was bought from Aladdin Chemistry Co. Ltd. (Shanghai, China). Bovine serum albumin (BSA) was purchased from Celartics biopharma Co. Ltd. 2-Morpholinoethanesulfonic acid (MES) was purchased from TCI Ltd. (Shanghai, China). All the above reagents were of analytical grade.

Sephacryl s-100 High Resolution was purchased by GE Healthcare Institute (Sweden). The phosphate-buffered saline (PBS) buffer was bought from Sangon Biotech (Shanghai, China) Co., Ltd. The BCA protein content kit was purchased from DingGuo Company (Beijing, China). The TIANamp Bacteria DNA Kit was obtained from Tiangen Bio (Beijing, China). Deionized water and *S. aureus* were from the School of Public Health, Jilin University.

### Synthesis of Fe_3_O_4_ MNPs

Previous methods were used to prepare Fe_3_O_4_ MNPs (32, 33). In brief, 1.08 g FeCl_3_·6H_2_O was dissolved in 20 ml of glycol for 10 min with magnetic stirring. Then, 1.2 g trisodium citrate dihydrate, 0.2 g sodium citrate, and 0.2 g PEG 6000 were added and stirred until completely dissolved. The mixture was transferred to a Teflon-lined stainless-steel autoclave and reacted at 200°C for 18 h. The product was washed with ethanol and distilled water three times and then dried under vacuum at 37°C for 12 h. The dried Fe_3_O_4_ MNPs were stored at 4°C and re-suspended with distilled water before use.

### Oriented Conjugation of IgY to Fe_3_O_4_ MNPs

The details of preparing IgY are described in Supporting Information.

To prepare the Fe_3_O_4_@IgY nanoprobe, 10 mg of MNPs were dissolved in MEST buffer (10 mM MES, 0.05% Tween20). The supernatant was discarded after magnetic separation, and 1 ml MEST was added for re-suspension. At room temperature for 30 min, 100 μl N-Hydroxysuccinimide and N-(3-dimethylamlnopropyl)-N'-ethylcarbodllmide hydrochloride (10 mg/ml) were added and the solution was mixed. After magnetic separation, the *S. aureus-*specific IgY solution was added and reacted at room temperature for 2 h. Then, 2% BSA solution was added and mixed for 1 h. The product was washed three times with deionized water. Finally, the obtained nanoprobe was suspended in 1 ml of deionized water and stored at 4°C for later use. The steps of random conjugation were the same, except that the MEST buffer was replaced with the PBST buffer (10 mM PBS, 0.05% Tween20, pH = 7.4).

### Bacteria Enrichment

All the bacterial strains used in this study were provided by the School of Public Health, Jilin University (Changchun, China), such as *Escherichia coli* O157:H7 (*E.coli* O157:H7, ATCC 25922), *Salmonella typhimurium* (*S. typhimurium*, ATCC 13311), *S. aureus* (ATCC 49775), and *Shigella Bogdii* (*S. Bogdii*, ATCC 9207).

*Staphylococcus aureus* was revived on Luria-Bertani agar (LA) plates and grown in the Luria-Bertani medium at 37°C for 18 h. The bacteria solution was diluted with sterile PBS buffer to 1 × 10^5^ CFU/ml. For the nanoprobe groups, 30 μl of 10 mg/ml nanoprobe was added to 100 μl bacteria solution, and the mixture was incubated at room temperature. The sterile PBS buffer was added to make the final volume of 1 ml. After enrichment for 40 min, magnetic separation and 50 μl of supernatant were coated on LA medium and cultured at 37°C for 18 h. The treatment of the positive group was the same, except that the nanoprobe was replaced by the PBS buffer.

The enrichment efficiency was calculated according to the equation:

Enrichment efficiency = (*N*_0_−*N*_s_)/*N*_0_

Where *N*_0_ and *N*_*s*_ are calculated from the positive group and nanoprobe groups, respectively.

### Reusability and Reproducibility

(i) Acid dissociation. After enrichment, remove the supernatant. Then, 1 ml of 1.5 M glycine-HCl, pH 2.5, was added and incubated for 60 min at 37°C, and then neutralized with 1.5 M Tris-HCl, pH 7.5 (34). Then, the enrichment step was the same as the description in ***Bacteria enrichment*.**

(ii) Reproducibility. The reproducibility was estimated by the intra-day and inter-day precision. The nanoprobe was stored at 4°C and the enrichment step was the same as described in ***Bacteria enrichment*.**

### Detection in Real Samples

The pork was purchased from the local supermarket. Then, 1 g of pork meat was ground and soaked in 19 ml of a sterile saline solution (0.9 % NaCl) overnight. The leachate was collected and filtered through a 0.22 μm microfilter (35). The detection procedure was as follows: the leachate was spiked with freshly cultured *S. aureu*s at different concentrations (from 1 × 10 to 1 ×10^6^ CFU/ml). The enrichment protocol was described in the section on bacteria enrichment, except that the PBS buffer was replaced by the leachate.

After enrichment, the bacteria were isolated by magnetic separation, and then the DNA was extracted and added to the real-time quantitative PCR system for amplification.

## Result and Discussion

### Characterization of Nanoprobe

**Figure 1:**
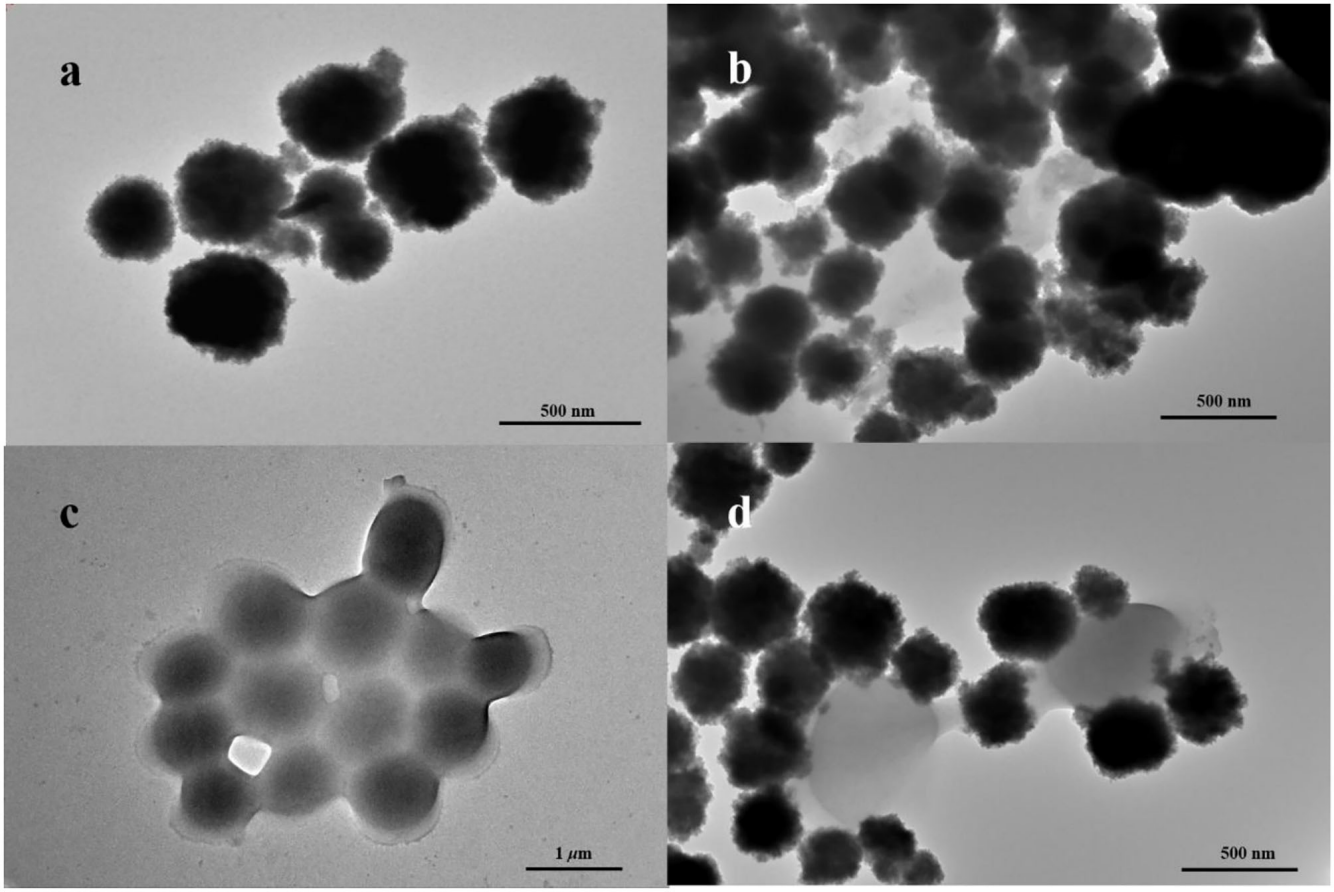
Transmission electron microscopy (TEM) images of **(a)** Fe_3_O_4_ MNPs, **(b)** nanoprobe, **(c)** *Staphylococcus aureus*, and **(d)** nanoprobe with *S. aureus*.

**Figure 2:**
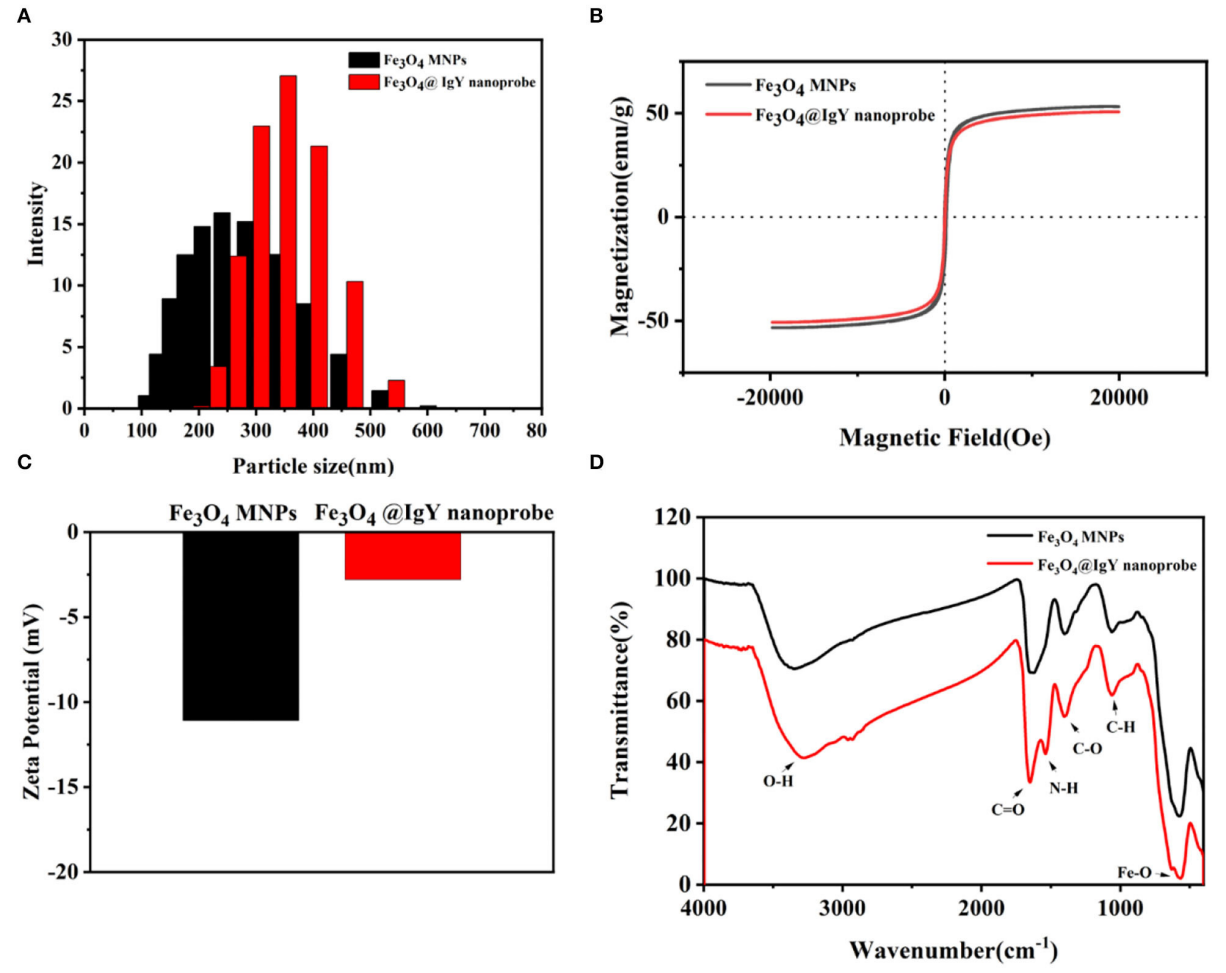
**(A)** Dynamic light scattering (DLS) spectra of Fe_3_O_4_ MNPs (black column) and nanoprobe (red column), **(B)** magnetic hysteresis loop of Fe_3_O_4_ MNPs (black line) and nanoprobe (red line), **(C)** zeta potential measurements of Fe_3_O_4_ MNPs and nanoprobe, and **(D)** Fourier-transform infrared (FITR) spectra of Fe_3_O_4_ MNPs and nanoprobe.

In this work, Fe_3_O_4_ MNPs were synthesized by the solvothermal method. Transmission electron microscopy (TEM) images showed that Fe_3_O_4_ MNPs were spherical (Figure 1a), with a mean diameter of about 242.45 ± 0.35 nm (Figure 2A). After conjugating with IgY, low-density shadows appear around the magnetic beads (Figure 1b), and the diameter increased to about 388.30 ± 0.14 nm (Figure 2A). It was because the IgY had been connected on the surface of MNPs. The synthesized nanoprobe was still well dispersed in the solution. As shown in Figure 2B, the magnetic saturation value of Fe_3_O_4_ MNPs and nanoprobe was 53.2 and 50.6 emu/g, respectively. The decrease in magnetic saturation indicated that IgY was conjugated on the MNPs. Moreover, the nanoprobe can be separated from the solution by an external magnet, indicating that the Fe_3_O_4_@IgY nanoprobe had a strong magnetic responsivity and was able to achieve efficient separation and enrichment of target bacteria in the solution. Figures 1c,d showed the good combination ability of nanoprobe with target bacteria.

The surface charges are usually changed when the material is modified with different functional groups. Zeta potential in deionized water was measured. Figure 2C shows that Fe_3_O_4_ MNPs performed a negative potential value, which can be attributed to the carboxy groups on the surface. After conjugating with IgY, the potentials of nanoprobe increased from −11.12 to −2.50 mV. The change resulted from the positively charged amino groups in IgY.

The Fourier-transform infrared (FT-IR) spectra of Fe_3_O_4_ MNPs and nanoprobe are shown in Figure 2D. In the spectrum of bare Fe_3_O_4_ MNPs, the characteristic band at 587, 1,050, 1,384, 1,640, and 3,310 cm^−1^ could be attributed to the stretching vibration of the Fe-O bond, C-N bond, C-H bond, C-O bond, C=O bond, and O-H bond, respectively (11, 36). After conjugation, the new peaks at 1,536 cm^−1^ were attributed to the N-H stretching vibration (37), indicating the occurrence of amino-carboxyl reactions on the surface of MNPs and the formation of peptide bonds. The results confirmed that the IgY was successfully bound to the surface of MNPs.

### Enrichment Efficiency of Nanoprobe

After the *S. aureus* was captured by the nanoprobe and separated, the remaining *S. aureus* in the supernatant was counted by plate coating.

**Figure 3:**
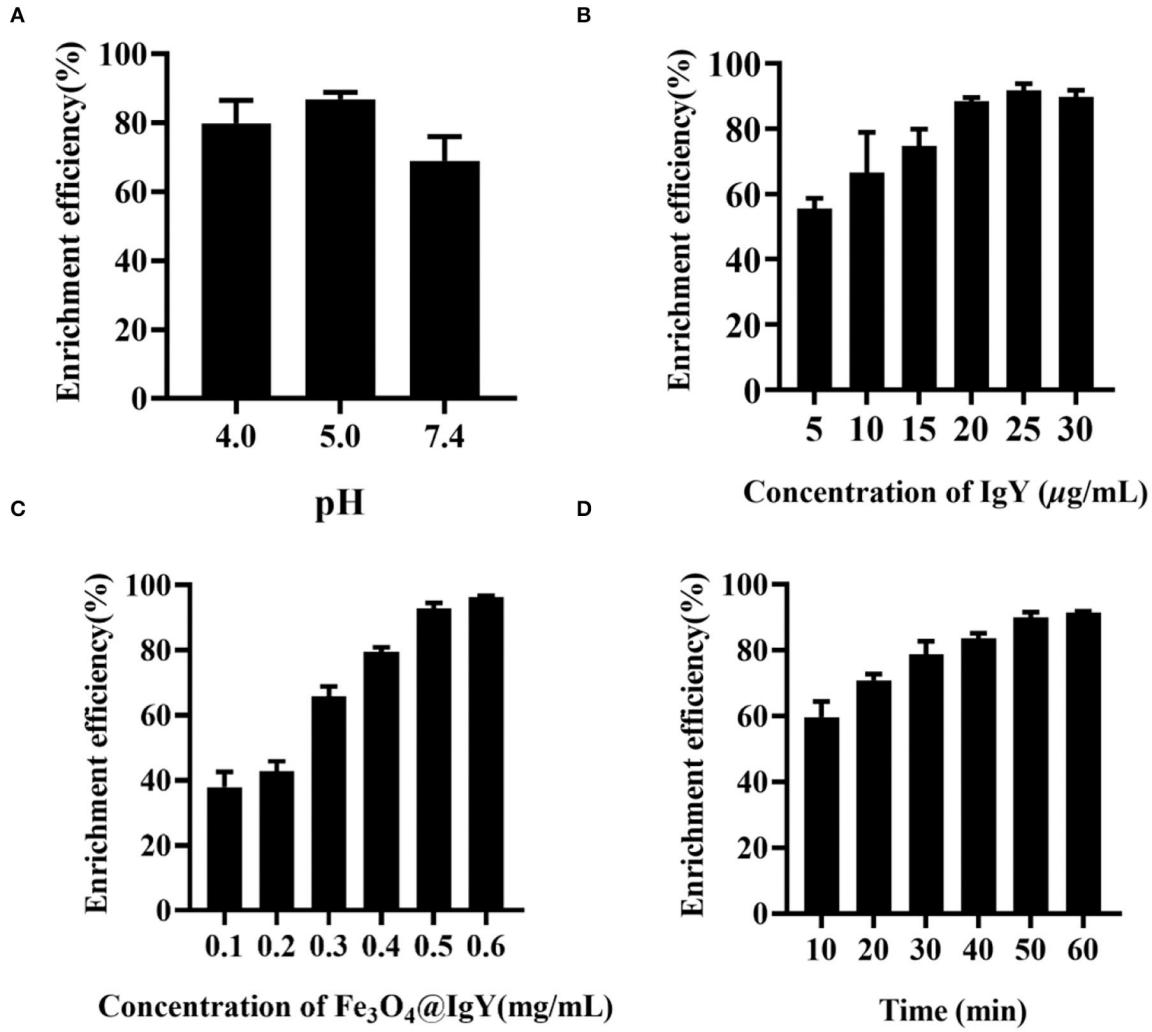
Enrichment efficiency of **(A)** different pH of buffer system, **(B)** concentration of IgY (5, 10, 15, 20, 25, and 30 μg/ml), **(C)** concentration of Fe_3_O_4_@IgY nanoprobe (0.1, 0.2, 0.3, 0.4, 0.5, and 0.6 mg/ml), and **(D)** enrichment time (10, 20, 30, 40, 50, and 60 min). Error bars represent the standard deviation (SD) of three replicates.

The results showed that pH was an important factor that affected the efficiency. Three kinds of nanoprobes were synthesized in different buffers (pH = 4.0, pH = 5.0, and pH = 7.4, respectively). As shown in Figure 3A, when the pH of the buffer system was 5.0, the enrichment efficiency of the nanoprobe was up to 89.1%, compared with 72.3% (pH = 7.4) and 79.5% (pH = 4.0) (Supplementary Figure S5). The enrichment results can be explained by the pI of IgY (Supplementary Figure S3). As shown in Supplementary Figure S3, the pI of IgY was between 5.96 and 6.25, and the fluctuation may be caused by the charge heterogeneity of IgY. When the pH of the buffer system was 5.0, lower than the pI of IgY, the Fc part of IgY was fully protonated. Based on the electrostatic effect, the IgY was oriented to the surface of the carboxylated Fe_3_O_4_ MNPs through the Fc part and then conjugated to form an immunomagnetic nanoprobe. When the pH of the buffer system was not optimal, IgY was not fully protonated and was randomly conjugated with Fe_3_O_4_ MNPs. Due to the covalent bonding of F_a_ and F_b_ parts with the carboxyl groups on the surface of the Fe_3_O_4_ MNPs, the ability of the nanoprobe to capture bacteria is reduced. Therefore, the MEST buffer (10 mM, 0.05% Tween20, pH = 5.0) was chosen as the optimal system for the nanoprobe synthesis and subsequent experiments.

### Reusability and Reproducibility

Acid dissociation was employed to evaluate the reusability of the nanoprobe. The immune complex was dissociated under acid buffer. Then, the enrichment efficiency (Supplementary Figure S8 and Supplementary Table S11) was compared to estimate the reusable. After acid dissociation, the efficiency decreased to 57.1%, indicating that the nanoprobe was not suitable for reuse. That may be due to the fact that once the specific immune complex was formatted, it was not easy to dissociate.

To evaluate the reproducibility, the intra-day and inter-day tests were estimated. As shown in Supplementary Figure S9, the enrichment efficiency of the nanoprobe shows good stability and reproducibility in 4 days. From the 5th day, the capture efficiency has greatly reduced. The decrease was largely related to the IgY failure. The reproducibility relied a lot on the titer of IgY, therefore, we suggested that the prepared nanoprobe was in a low-temperature environment and used within 4 days after being prepared.

### Evaluation of Enrichment System

For better enrichment efficiency, several experimental conditions were optimized, such as IgY concentration, Fe_3_O_4_@IgY nanoprobe concentration, and enrichment time. By comparing the enrichment efficiency and TGA result under different parameters (Figure 3, Supplementary Figure S4, Supplementary Tables S2–4, S7–10), the followings were selected as the optimal conditions (a) IgY concentration: 25 μg/ml, (b) Fe_3_O_4_@IgY nanoprobe concentration: 0.5 mg/ml, and (c) enrichment time: 50 min. Under the optimal conditions, the enrichment efficiency of the oriented nanoprobe was 92.8%, and the random conjugation was 81.7% (Supplementary Table S5).

To investigate the specificity of the nanoprobe, interference bacteria were added to the enrichment system, such as *E.coli* O157:H7, *S. typhimurium, S. aureus*, and *S. Bogdii*. The enrichment efficiency of interference bacteria was between 2.5 and 17.5% (Supplementary Table S6 and Supplementary Figure S6), indicating that the synthesized nanoprobe can only identify the *S. aureus*, showing good specificity. The specificity was mostly attributed to the well-prepared IgY, and the results indicated that the IgY had good antigen recognition characteristics similar to IgG. Conjugated with nanoparticles, IgY still exhibited a good specificity, providing a novel idea for its wide application.

The applicability of the prepared nanoprobe was further explored by combining the nanoprobe with real-time quantitative PCR. The limit of detection (LOD) with enrichment using the prepared nanoprobe was 10^3^ CFU/ml, which was lower than the LOD of real-time quantitative PCR (Supplementary Figure S7) and many other strategies (Table 1).

**Table 1:** Comparison of the method with previous methods.

**Method**	**Detection time (h)**	**LOD(CFU)**	**References**
MNP-TiO_2_-AP-SMCC	5	3.7 × 10^2^	(38)
IMS-PMA-MPCR	2	8.4 × 10^3^	(39)
Droplet digital PCR	4	2.9 × 10^3^	(40)
Heptaplex PCR assay	4	1 × 10^3^	(41)
Oriented conjugation Fe_3_O_4_@IgY nanoprobe with PCR	2	1 × 10^3^	This study

These results confirmed that the nanoprobe showed good enrichment efficiency in the real sample. When combined with PCR, the LOD significantly decreased by two orders of magnitude. However, the nanoprobe still has some limitations. The biggest defect was that our nanoprobe cannot be reused. Due to the stable binding of bacteria and IgY, the capture efficiency decreased sharply after acid dissociation. Although the cost of material synthesis was low and easy to synthesize, the reusable nanoprobe, serving as an eco-friendly microbiological detection reagent, has great application prospects. Second, the prepared nanoprobe cannot be stored for a long time. The capture efficiency can be stable and satisfactory only for 4 days. Therefore, if the nanoprobe is applied to a wider range of fields, it must be committed to develop reusable probes and further improving the stability in complex environments.

## Conclusion

In this article, the oriented conjugation Fe_3_O_4_@IgY nanoprobe was prepared based on the pH adjusting. Due to the adequate exposure of antigen recognition epitope, the enrichment efficiency toward target bacteria was up to 92.8%. The excellent enrichment capacity was also confirmed in the real sample, showing a decrease in LOD. Besides, the feasible oriented conjugation strategy of IgY provided a new idea of site-specific conjugation for protein with a similar structure.

## Data Availability Statement

The original contributions presented in the study are included in the article/Supplementary Material, further inquiries can be directed to the corresponding author/s.

## Author Contributions

JW and HuL: conceptualization, funding acquisition, and project administration. JW and CZ: methodology and supervision. HS: software. JW: validation. XS and HaL: formal analysis and writing—original draft preparation. HS and JJ: investigation. XS: resources. XS and SW: data curation and visualization. HS, CZ, and SW: writing—review and editing. All authors have read and agreed to the published version of the manuscript.

## Funding

This research was funded by Jilin Province Science and Technology Development Plan Item, grant number 20200403035SF.

## Conflict of Interest

The authors declare that the research was conducted in the absence of any commercial or financial relationships that could be construed as a potential conflict of interest.

## Publisher's Note

All claims expressed in this article are solely those of the authors and do not necessarily represent those of their affiliated organizations, or those of the publisher, the editors and the reviewers. Any product that may be evaluated in this article, or claim that may be made by its manufacturer, is not guaranteed or endorsed by the publisher.
