# Supplementary material for: Preparation of IgY Oriented Conjugated Fe3O4 MNPs as Immunomagnetic Nanoprobe for Increasing Enrichment Efficiency of Staphylococcus aureus Based on Adjusting the pH of the Solution System
Source: Front Public Health. 2022 May 20;10:865828. doi: 10.3389/fpubh.2022.865828 (PMC9163370; doi:10.3389/fpubh.2022.865828)
Supplement: Supplementary file 1 [file Data_Sheet_1.pdf]

Supplementary Material

1 Extraction, determination, and purification of specific IgY towards *Staphylococcus aureus.*

Extraction: Break the egg, and separate the yolk fluid. Add 2 volumes of PBS buffer and 3.5% PEG 6000 (w/v), shake and mix for 10 minutes, and centrifuge at 10,000 rpm for 20 minutes. After the supernatant was filtered, record the volume of the supernatant, add 8.5% PEG 6000 (w/v), shake and mix again and centrifuge. Discard the supernatant, add 12% PEG 6000 (w/v), shake and mix again and centrifuge. The supernatant was discarded and the precipitate was IgY. It was resuspended in 1ml PBS, dialyzed overnight with 0.1% saline, and then dialyzed with PBS buffer for 4 hours. The collected solution was stored at -20 °C.

Protein content determination: The BCA protein content kit was used to determine the protein content of the collected IgY. Configure the protein standard series according to the kit instructions. Dilute the IgY appropriately, take 10 μL and add to a 96-well plate. Add BCA working solution, then put the plate in a 37 ℃ oven, and incubate for 30 minutes. After the reaction, the OD 562 nm was measured with a microplate reader. The standard curve was drawn with the protein standard series as the abscissa and OD 562 nm as the ordinate, and the protein concentration of the sample was calculated according to the standard curve. Fig.S1. is the obtained standard curve. According to the standard curve, the protein concentration of IgY was 28.6 mg/mL.

ELISA to determine titer: 1×10^8 CFU/mL *Staphylococcus aureus* was used as antigen to coat the 96-well plate. BSA solution was used as blank control, and the concentration was consistent with the dilution gradient of IgY. When the ratio of the sample OD 450 value (P) to the negative control OD 450 nm value (N) P/N ≥ 2.1, the maximum dilution factor of the sample required is the antibody titer. After calculation, the titer of IgY is 1:128000.

Gel filtration chromatography purification: Acryl dextran gel s-100hr was used as the packing material for purification, and the column size was 1.6×40 cm. Use PBS buffer as the eluent, after equilibrating the column, add 1 mL IgY solution to start the elution. Collect the sample every 15 mL in a tube, and determine the protein content of the collected sample.

SDS-PAGE to identify the purity: Use SDS-PAGE kit to identify the purity of IgY before and after purification. Figure S2 shows the result of electrophoresis. The target heavy chain of the IgY is about 70 kD, and the light chain is about 25 kD.

2 Real-time quantitive PCR.

Real-time quantitive PCR(RT-PCR) was performed after DNA extraction.The RT-PCR mixture included 2 μL DNA, 1 μL each of the two primers (10 μmol)(Table S1)[1], 12.5 μL SYBR Green Mix, and 8.5 μL distilled water. The PCR process was consisted of DNA denaturation at 95 °C for 5 min, followed by 40 cycles of 95 °C for 30 sec, 55°C for 30 sec, and 72°C for 30 sec, with a final extension at 72°C for 5 min. The *S.aureus* DNA extracted in previous work was used as positive control, and the distilled water was used as negative control. All the steps was the same, except the DNA in mixture was replaced by the *S.aureus* DNA and distilled water. The results of RT-PCR was

analyzed by Applied Biosystems of QuantStudio 3 (Thermo Fisher Scientific, USA). The Ct Threshold was used to determine the specific amplification.

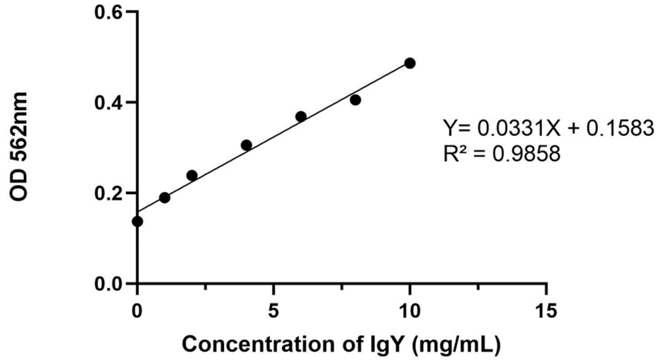

Fig. S1. Linear plots of OD 562 nm versus IgY concentration

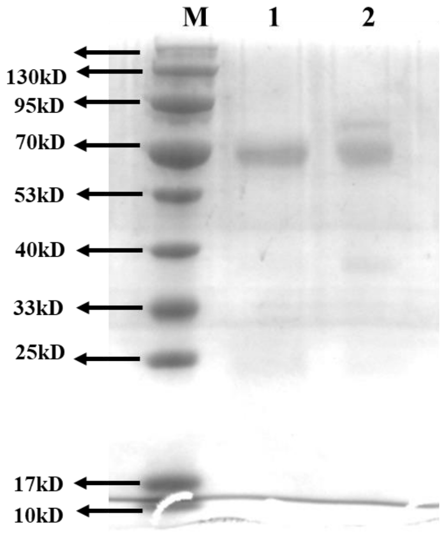

Fig. S2. SDS-PAGE, M: Marker, lane 1: IgY after purification, lane 2: mixture before purification.

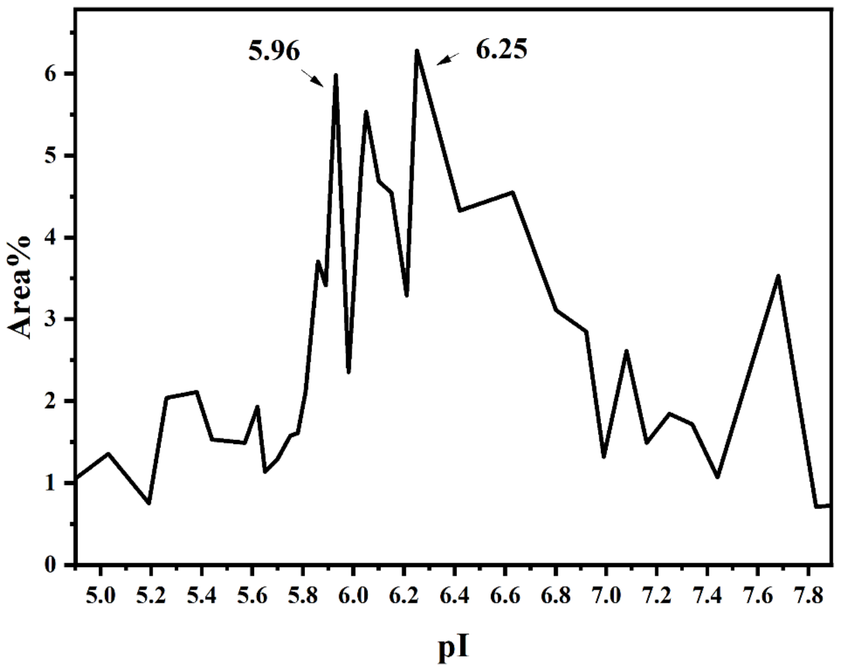

Fig. S3. Isoelectric point(pI) test of IgY.

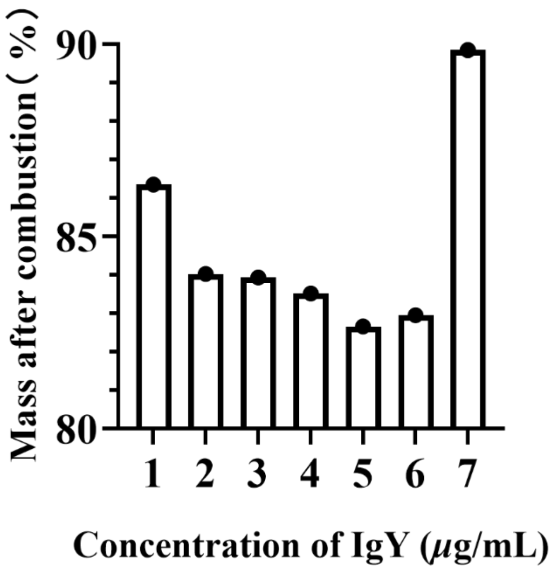

Fig. S4. TGA results.(1→6: 5, 10, 15, 20, 25, 30 μg/mL, pH = 5.0;7: 25 μg/mL, pH = 7.4)

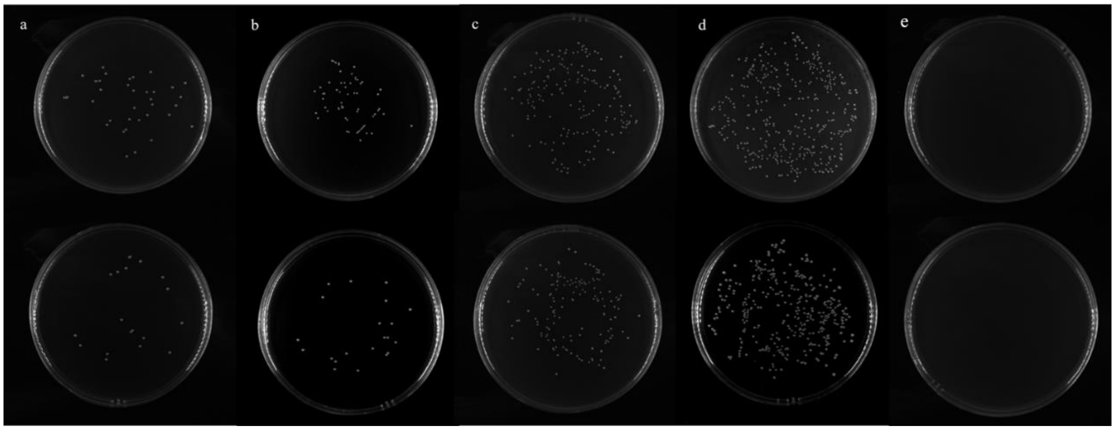

Fig. S5. Bacteria culture. (**a**)pH=4.0, (**b**) pH=5.0, (**c**) pH=7.4, (**d**) positive control, (**e**) negative control.

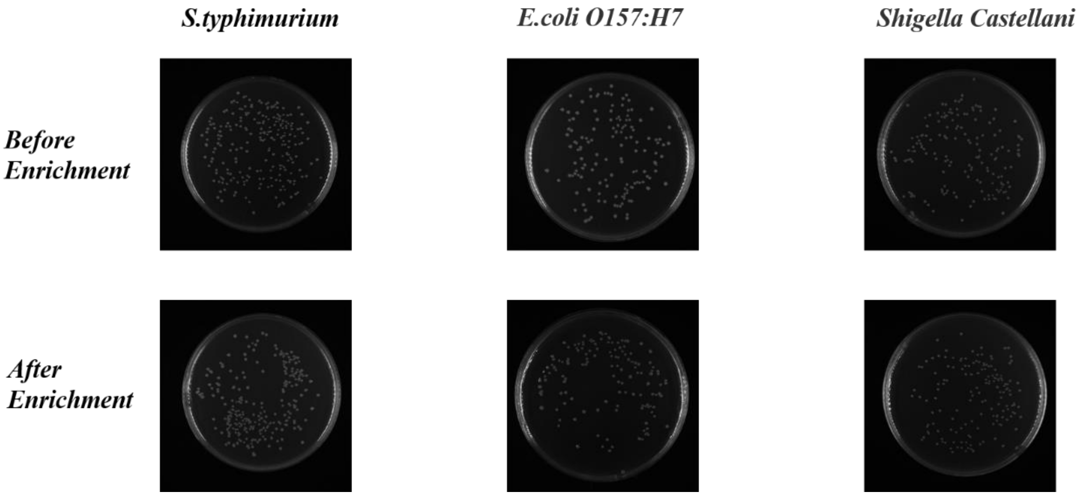

Fig. S6. Selectivity of nanoprobe.

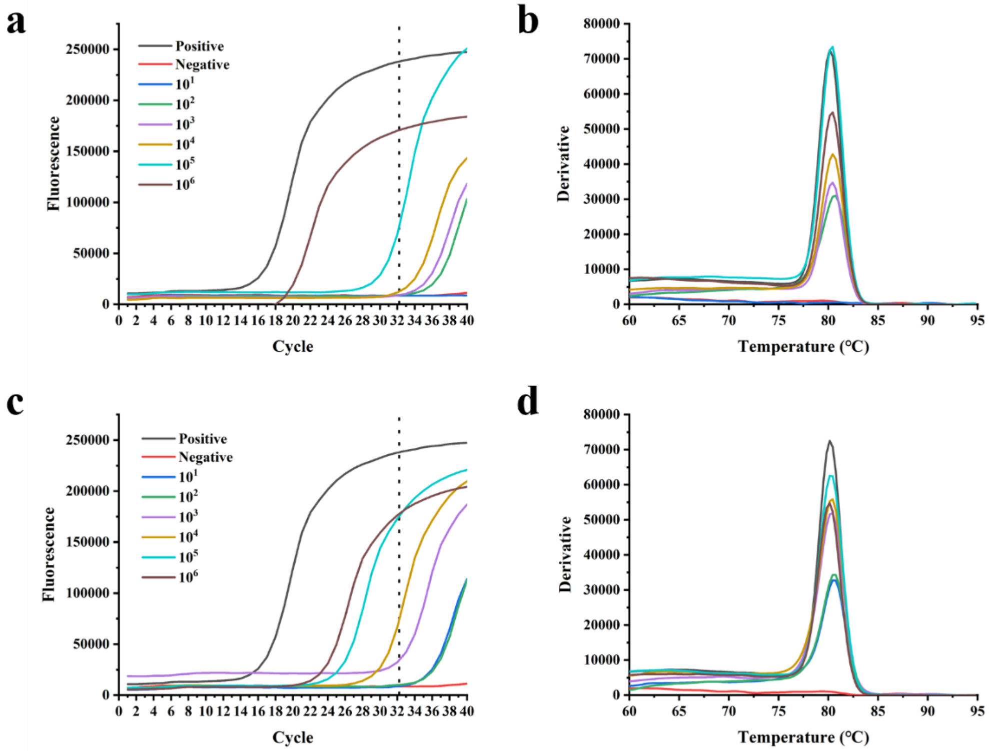

Fig. S7. Real-time quantitative PCR of *S. aureus* (10^1 ~ 10^6 CFU/mL) in real sample (**a)** without enrichment(Ct Threshold = 32.21) and melt curve**(b)**;(**c)** with enrichment(Ct Threshold = 32.21) and melt curve**(d)**. The Ct Threshold was marked with black dotted line in figure.

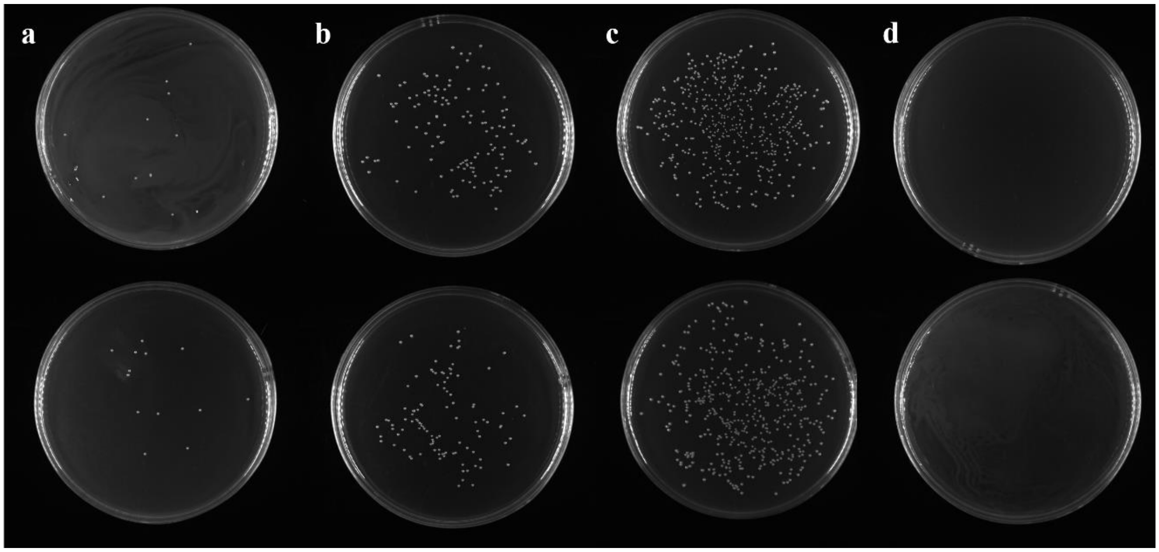

Fig. S8. Reusability test(a)nanoprobe (b)nanoprobe after acid dissociation (c)positive control (d) negative control.

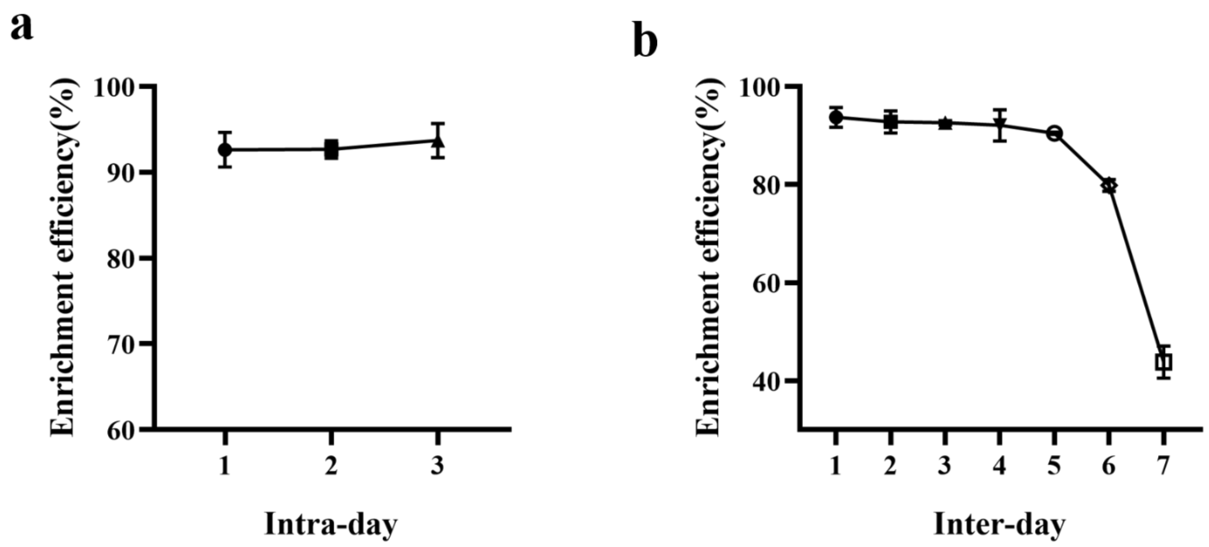

Fig. S9. Reproducibility test(a)Intra-day (b)Inter-day.

Table.S1. The sequence of PCR primer.

	Sequence(5'-3')
Nuc-f	C C T G A A G C A A G T G C A T T T A C G A
Nuc-r	C T T T A G C C A A G C C T T G A C G A A C T

Table.S2. Efficiency of different concentration of IgY (μg/mL)*(* $\bar{x}$ *±s, n=3)*.

Concentration of IgY (μg/mL)	5	10	15	20	25	30
N_0	48.8±1.5					
N_s	21.6±1.2	16.3±4.9	12.3±2.1	5.7±0.5	4.0±0.8	5.0±0.8
Enrichment efficiency (%)	55.6	66.5	74.7	88.4	91.8	89.7

Table.S3. Efficiency of different concentration of Fe_3O_4@IgY (mg/mL) *(* $\bar{x}$ *±s, n=3)*.

Concentration of of Fe_3O_4@IgY (mg/mL)	0.1	0.2	0.3	0.4	0.5	0.6
N_0	269.3±11.1					
N_s	167.0±10.2	154.0±6.7	92.0±6.7	55.0±2.9	19.3±3.8	10.0±0.8
Enrichment efficiency（%）	38.0	42.8	65.8	79.6	92.8	96.3

Table.S4. Efficiency of different enrichment time($\bar{x}$ ±*s, n=3*).

Enrichment time (min)	10	20	30	40	50	60
N_0	254.0±5.7					
N_s	102.3±9.9	74.0±4.1	54.0±8.3	41.7±3.4	25.3±3.3	25.0±3.3
Enrichment efficiency（%）	59.7	70.9	78.7	83.6	90.0	90.2

Table.S5. Efficiency of different pH of buffer system at optimal condition($\bar{x}$ ±*s, n=3*).

pH of buffer system	5.0	7.4
N_0	269.3±11.1	153.3±4.7
N_s	19.3±3.8	28.0±9.5
Enrichment efficiency（%）	92.8	81.7

Table.S6. Efficiency of interference bacteria($\bar{x}$ ±*s, n=3*).

Bacterial species	*S.typhimurium*	*E.coli O157:H7*	*Shigella Castellani*
N_0	287.3±3.4	153.3±7.6	106.0±8.6

N_s	237.0±3.5	136.3±3.4	103.3±2.5
Enrichment efficiency（%）	17.5	11.1	2.5

Table.S7. Statistical analysis(EE* of different pH buffer systems)

	Sum of Squares	df	Mean Square	F	Sig.
Between Groups	484.389	2	242.194	7.598	.023
Within Groups	191.267	6	31.878		
Total	675.656	8			

*EE: enrichment efficiency

Table.S8. Statistical analysis (Fe_3O_4@IgY nanoprobe concentration, 0.5 versus 0.6 mg/mL)

		Levene's Test for Equality of Variances		t-test for Equality of Means		
		F	Sig.	t	df	Sig. (2-tailed)
EE*	Equal variances assumed	4.430	.103	-1.840	4	.140
	Equal variances not assumed			-1.840	2.685	.174

*EE: enrichment efficiency

Table.S9. Statistical analysis (Enrichment time, 50 versus 60 min)

		Levene's Test for Equality of Variances		t-test for Equality of Means		

		F	Sig.	t	df	Sig. (2-tailed)
EE*	Equal variances assumed	2.784	.171	-1.488	4	.211
	Equal variances not assumed			-1.488	2.347	.257

*EE: enrichment efficiency

Table.S10. Optimization of the conditions.

Parameter	*P* value	Optimal condition
pH buffer systems	0.023	5.0
Concentration of of Fe_3O_4@IgY	0.14	0.5 mg/mL
Enrichment time	0.21	50 min

Table.S11. Efficiency before and after acid dissociation($\bar{x}$ ±*s*, *n*=3).

Group	nanoprobe	nanoprobe after acid dissociation
N_0	257.3 ± 11.0	
N_s	17.0 ± 2.6	110.3 ± 14.7
Enrichment efficiency（%）	93.4	57.1

Reference:

[1] He, S., Huang, Y., Ma, Y., Yu, H., Pang, Bo, Liu, X., Yin, C., Wang, X., Wei, Y., Tian, Y., Zhao, C., Xu, K., Wang, J., Lv, C., Song, X., & Jin, M. (2022). Detection of four foodborne pathogens based on magnetic separation multiplex PCR and capillary electrophoresis. Biotechnol. J., 17, e2100335.
